# Sparse graphical Gaussian modeling of the isoprenoid gene network in *Arabidopsis thaliana*

**DOI:** 10.1186/gb-2004-5-11-r92

**Published:** 2004-10-25

**Authors:** Anja Wille, Philip Zimmermann, Eva Vranová, Andreas Fürholz, Oliver Laule, Stefan Bleuler, Lars Hennig, Amela Prelić, Peter von Rohr, Lothar Thiele, Eckart Zitzler, Wilhelm Gruissem, Peter Bühlmann

**Affiliations:** 1 Reverse Engineering Group, Swiss Federal Institute of Technology (ETH), Zurich; 2 Colab, ETH, Zurich 8092, Switzerland; 3 Seminar for Statistics, ETH, Zurich 8092, Switzerland; 4 Institute for Plant Sciences and Functional Genomics Center Zurich, ETH, Zurich 8092, Switzerland; 5 Computer Engineering and Networks Laboratory, ETH, Zurich 8092; 6 Institute of Computational Science, ETH, Zurich 8092, Switzerland

## Abstract

We present a novel graphical Gaussian modeling approach for reverse engineering of genetic regulatory networks with many genes and few observations. When applying our approach to infer a gene network for isoprenoid biosynthesis in *Arabidopsis thaliana*, we detect modules of closely connected genes and candidate genes for possible cross-talk between the isoprenoid pathways. Genes of downstream pathways also fit well into the network. We evaluate our approach in a simulation study and using the yeast galactose network.

## Abstract 2

A novel approach for modeling gene-regulatory networks, based on graphical Gaussian modeling, is used to create a network for the isoprenoid biosynthesis pathway in *Arabidopsis*.

## Background

The analysis of genetic regulatory networks has received a major impetus from the huge amounts of data made available by high-throughput technologies such as DNA microarrays. The genome-wide, massively parallel monitoring of gene activity will increase the understanding of the molecular basis of disease and facilitate the identification of therapeutic targets.

To fully uncover regulatory structures, different analysis tools for transcriptomic and other high-throughput data will have to be used in an integrative or iterative fashion. In simple eukaryotes or prokaryotes, gene-expression data has been combined with two-hybrid data [1] and phenotypic data [2] to successfully predict protein-protein interaction and transcriptional regulation on a large scale. If the principal organization of a gene network has been established, differential equations may be used to study its quantitative behavior [3,4].

In higher organisms, however, little is known about regulatory control mechanisms. As a first step in reverse engineering of genetic regulatory networks, structural relationships between genes can be explored on the basis of their expression profiles. Here, we focus on graphical models [5,6] as a probabilistic tool to analyze and visualize conditional dependencies between genes. Genes are represented by the vertices of a graph and conditional dependencies between their expression profiles are encoded by edges. Graphical modeling can be carried out with directed and undirected edges, with discretized and continuous data. Over the past few years, graphical models, in particular Bayesian networks, have become increasingly popular in reverse engineering of genetic regulatory networks [7-10].

Graphical models are powerful for a small number of genes. As the number of genes increases, however, reliable estimates of conditional dependencies require many more observations than are usually available from gene-expression profiling. Furthermore, because the number of models grows super-exponentially with the number of genes, only a small subset of models can be tested [10]. Most important, a large number of genes often entails a large number of spurious edges in the model [11]. The interpretation of the graph within a conditional-independence framework is then rendered difficult [12]. Even a search for local dependence structures and subnetworks with high statistical support [7] provides no guarantee against the detection of numerous spurious features.

Some of these problems may be circumvented by restricting the number of possible models or edges [10,13] or by exploiting prior knowledge on the network structure. So far, however, this prior knowledge is difficult to obtain.

As an alternative approach to modeling genetic networks with many genes, we propose not to condition on all genes at a time. Instead, we apply graphical modeling to small subnetworks of three genes to explore the dependence between two of the genes conditional on the third. These subnetworks are then combined for making inferences on the complete network. This modified graphical modeling approach makes it possible to include many genes in the network while studying dependence patterns in a more complex and exhaustive way than with only pairwise correlation-based relationships.

For an independent validation of our method, we compare our modified graphical Gaussian modeling (GGM) approach with conventional graphical modeling in a simulation study. We show at the end of the Results section that our approach outperforms the standard method in simulation settings with many genes and few observations. For a further evaluation with real data, we apply our approach to the galactose-utilization data from [14] to detect galactose-regulated genes in *Saccharomyces cerevisiae*.

The main aim of this methodological work, however, was to elucidate the regulatory network of the two isoprenoid biosynthesis pathways in *Arabidopsis thaliana *(reviewed in [15]). The greater part of this paper is therefore devoted to the inference and biological interpretation of a genetic regulatory network for these two pathways. To motivate our novel modeling strategy, we first describe the problems that we encountered with standard GGMs before presenting the results of our modified GGM approach.

## Results

Isoprenoids serve numerous biochemical functions in plants: for example, as components of membranes (sterols), as photosynthetic pigments (carotenoids and chlorophylls) and as hormones (gibberellins). Isoprenoids are synthesized through condensation of the five-carbon intermediates isopentenyl diphosphate (IPP) and dimethylallyl diphosphate (DMAPP). In higher plants, two distinct pathways for the formation of IPP and DMAPP exist, one in the cytosol and the other in the chloroplast. The cytosolic pathway, often described as the mevalonate or MVA pathway, starts from acetyl-CoA to form IPP via several steps, including the intermediate mevalonate (MVA). In contrast, the plastidial (non-mevalonate or MEP) pathway involves condensation of pyruvate and glyceraldehyde 3-phosphate via several intermediates to form IPP and DMAPP. Whereas the MVA pathway is responsible for the synthesis of sterols, sesquiterpenes and the side chain of ubiquinone, the MEP pathway is used for the synthesis of isoprenes, carotenoids and the side chains of chlorophyll and plastoquinone. Although both pathways operate independently under normal conditions, interaction between them has been repeatedly reported [16,17].

Reduced flux through the MVA pathway after treatment with lovastatin can be partially compensated for by the MEP pathway. However, inhibition of the MEP pathway in seedlings leads to reduced levels in carotenoids and chlorophylls, indicating a predominantly unidirectional transport of isoprenoid intermediates from the chloroplast to the cytosol [16,18], although some reports indicate that an import of isoprenoid intermediates into the chloroplast also takes place [19-21].

### Application of standard GGM to isoprenoid pathways in *Arabidopsis thaliana*

To gain more insight into the cross-talk between both pathways at the transcriptional level, gene-expression patterns were monitored under various experimental conditions using 118 GeneChip (Affymetrix) microarrays (see Additional data files 1 and 2). To construct the genetic regulatory network, we focused on 40 genes, 16 of which were assigned to the cytosolic pathway, 19 to the plastidal pathway and five encode proteins located in the mitochondrion. These 40 genes comprise not only genes of known function but also genes whose encoded proteins displayed considerable homology to proteins of known function. For reference, we adopt the notation from [22] (see Table 1).

**Figure 1:**
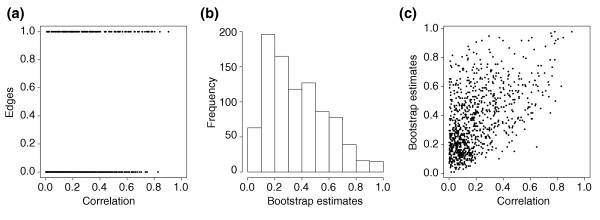
Bootstrapped GGM of the isoprenoid pathway. **(a) **Comparison between absolute pairwise correlation coefficients and presence of edges. Dots at 0 and 1 denote absent and present edges respectively. **(b) **Histogram of the bootstrap edge probabilities. **(c) **Comparison between absolute pairwise correlation coefficients and bootstrap edge probabilities for all 780 possible edges.

**Figure 2:**
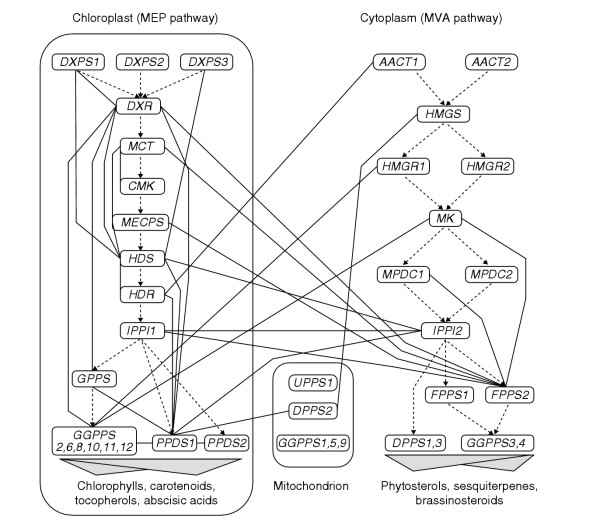
Bootstrapped GGM of the isoprenoid pathway with a cutoff at 0.8. The solid undirected edges connecting individual genes (in boxes) represent the GGM. Dotted directed edges mark the metabolic network, and are not part of the GGM. The grey shading indicates metabolic links to downstream pathways.

The genetic-interaction network among these genes was first constructed using GGM with backward selection under the Bayesian information criterion (BIC) [23]. This was carried out with the program MIM 3.1 [24] (see Materials and methods for further details). The network obtained had 178 (out of 780) edges - too many to single out biologically relevant structures. Therefore, bootstrap resampling was applied to determine the statistical confidence of the edges in the model (Figure 1b). For the bootstrap edge probabilities, only a cutoff level as high as 0.8 led to a reasonably low number of selected edges (31 edges, Figure 2). However, a comparison between bootstrap-edge probabilities and the pairwise correlation coefficients suggested that for such a high cutoff level, many true edges may be missed. For example, the gene *AACT2 *appears to be completely independent from all genes in the model although it is strongly correlated with *MK*, *MPDC1 *and *FPPS2 *(see Additional data file 4 for the correlation patterns).

This phenomenon had already been observed in a simulation study by Friedman *et al*. [25] and may be related to the surprisingly frequent appearance of edges with a low absolute pairwise correlation coefficient but a high bootstrap estimate (Figure 1c). Although there is no concise explanation for this pattern, one conjecture would be that the simultaneous conditioning on many variables introduces many spurious edges with little absolute pairwise correlation but high absolute partial correlation into the model. Our modification for GGMs is to improve upon this drawback.

### Application of our modified GGM approaches

As described in more detail in Materials and methods, our approach aims at modeling dependencies between two genes by taking the effect of other genes separately into account. In the hope of identifying direct co-regulation between genes, an edge is drawn between two genes *i *and *j *when their pairwise correlation is not the effect of a third gene. Each edge has therefore a clear interpretation.

We have developed two versions of our method: a frequentist approach in which each edge is tested for presence or absence; and a likelihood approach with parameters *θ*_*ij*_, which describe the probability for an edge between *i *and *j *in a latent random graph. One main benefit of the second version over full graphical models is that one can easily test on a large scale how well additional genes can be incorporated into the network. This allows the selection of additional candidate genes for the network in a fast and efficient way.

**Figure 3:**
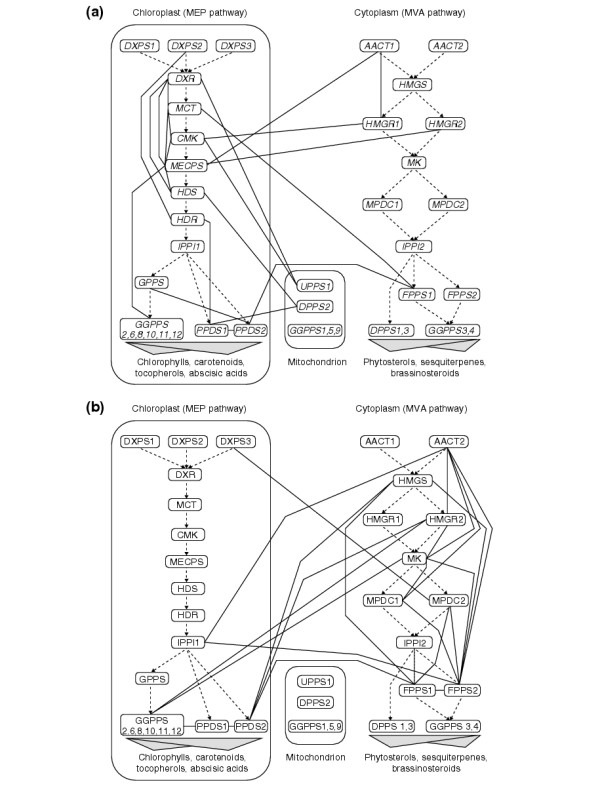
Dependencies between genes of the isoprenoid pathways according to the frequentist modified GGM method. **(a) **Subgraph of the gene module in the MEP pathway; **(b) **subgraph of the gene module in the MVA pathway. For an explanation of what the edges and shading indicate see legend to Figure 2.

We have applied and tested our modified GGM approaches by constructing a regulatory network of the 40 genes in the isoprenoid pathways in *A. thaliana *and by attaching 795 additional genes from 56 other metabolic pathways to it. Figure 3 shows the network model obtained from the frequentist modified GGM approach. Because we find a module with strongly interconnected genes in each of the two pathways, we split the graph into two subgraphs, each displaying the subnetwork of one module and its neighbors. Our finding provides a further example that within a pathway many consecutive or closely positioned genes are potentially jointly regulated [26].

In the MEP pathway, the genes *DXR*, *MCT*, *CMK *and *MECPS *are nearly fully connected (upper panel of Figure 3). From this group of genes, there are a few edges to genes in the MVA pathway. Among these genes, *AACT1 *and *HMGR1 *form candidates for cross-talk between the MEP and the MVA pathway because they have no further connection to the MVA pathway. Their correlation to *DXR*, *MCT*, *CMK *and *MECPS *is always negative.

Similarly, the genes *AACT2*, *HMGS*, *HMGR2*, *MK*, *MPDC1*, *FPPS1 *and *FPPS2 *share many edges in the MVA pathway (lower panel of Figure 3). The subgroup *AACT2*, *MK*, *MPDC1 *and *FPPS2 *is completely interconnected. From these genes, we find edges to *IPPI1 *and *GGPPS12 *in the MEP pathway. Whereas *IPPI1 *is positively correlated with *AACT2*, *MK*, *MPDC1 *and *FPPS2*, *GGPPS12 *displays negative correlation to the four genes.

**Figure 4:**
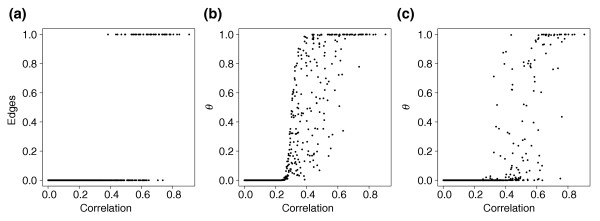
Comparison of the absolute pairwise correlation coefficients and the modified GGM approaches. **(a) **Selected edges in the frequentist modified GGM approach (0 and 1 denote absent and present edges respectively). **(b) ***θ*-values in the latent random graph approach. **(c) ***θ*-values after attaching 795 genes from other pathways.

In contrast to the conventional graphical model, we could now identify the connection between *AACT2 *and *MK*, *MPDC1 *and *FPPS2*. In general, we found a better agreement between the absolute pairwise correlation and the selected edges (frequentist approach) or the probability parameters *θ *(latent random graph approach). Figures 4a and 4b show the selected edges and *θ*-values as a function of the absolute pairwise correlation.

### Attaching additional pathway genes to the network

Following construction of the isoprenoid genetic network, 795 additional genes from 56 metabolic pathways were incorporated. Among these were genes from pathways downstream of the two isoprenoid biosynthesis pathways, such as phytosterol biosynthesis, mono- and diterpene metabolism, porphyrin/chlorophyll metabolism, carotenoid biosynthesis, plastoquinone biosynthesis for example. Using the second version of our method, that is, the latent random graph approach, we compared *θ*-values for all gene pairs in the network with and without attaching these additional genes (Figure 4b and 4c). As expected, the parameters *θ *for the edge probabilities decreased if additional genes were included in the isoprenoid network (see Materials and methods). After addition, if for a gene pair *i*, *j*, *θ*_*ij *_dropped by more than 0.3, it was assumed that the dependence between *i *and *j *could be 'explained' by some of the additional genes.

To find these genes out of all additionally tested candidates *k*, GGMs with genes *i*, *j *and *k *were formed. A gene *k *was considered to explain the dependency between *i *and *j *when an edge between *i *and *j *was not supported in the GGM, that is, when the null hypothesis *ρ*_*ij*|*k *_= 0 was accepted in the corresponding likelihood ratio test. *k *was then taken to 'attach well' to the gene pair *i*, *j*.

Thus, for each gene pair *i*, *j *whose parameter *θ*_*ij *_dropped by more than 0.3, we obtained a list of well-attaching genes. Genes appearing significantly frequently in these lists of well-attaching genes were assumed to connect well to the complete genetic network. We tested for significance by randomization: For each gene pair *i*, *j*, a randomized list of well-attaching genes was formed with the same size as the original gene list. To explore which pathways attach significantly well to the MVA and MEP pathways, the portion of genes from each of the 56 pathways was summed over all gene pairs *i*, *j*. These sums were then compared for the originally attached genes and the sums of randomly attached genes in 100 datasets.

Table 2 shows the pathways whose genes were found to attach significantly frequently to the MVA pathway, the MEP pathway, or both pathways. Interestingly, from all 56 metabolic pathways considered, we predominantly find that genes from downstream pathways fit well into the isoprenoid network. These results suggest a close regulatory connection between isoprenoid biosynthesis genes and groups of downstream genes. On the one hand, we find strong connections between the MEP pathway and the plastoquinone, the carotenoid and the chlorophyll pathways (experimentally supported by [15,16,27]). On the other hand, the plastoquinone and phytosterol biosynthesis pathways appear to be closely related to the genetic network of the MVA pathway.

On a metabolic level, our results are substantiated by earlier labeling experiments using [1-^13^C] glucose, which revealed that sterols were formed via the MVA pathway, while plastidic isoprenoids (β-carotene, lutein, phytol and plastoquinone-9) were synthesized using intermediates from the MEP pathway [27]. Moreover, incorporation of [1-^13^C]- and [2,3,4,5-^13^C_4_]1-deoxy-D-xylulose into β-carotene, lutein and phytol indicated that the carotenoid and chlorophyll biosynthesis pathways proceed from intermediates obtained via the MEP pathway [28].

In contrast, a close connection between the MVA and the MEP pathways could not be detected. This suggests that cross-talk on the transcriptional level may be restricted to single genes in both pathways.

In a further analysis step, we examined which gene pairs the four identified pathways (plastoquinone, carotenoid, chlorophyll, and phytosterols) attached to. Genes from the plastoquinone pathway were predominantly linked to the genes *DXR*, *MCT*, *CMK*, *GGPPS11*, *GGPPS12*, *AACT1*, *HMGR1 *and *FPPS1*, supporting the hypothesis that *AACT1 *and *HMGR1 *are involved in communication between the MEP and MVA pathways.

Genes from the carotenoid pathway attached to *DXPS2*, *HDS*, *HDR*, *GGPPS11*, *DPPS2 *and *PPDS2*, whereas the chlorophyll biosynthesis appears to be related to *DXPS2*, *DXPS3*, *DXR*, *CMK*, *MCT*, *HDS*, *HDR*, *GGPPS11 *and *GGPPS12*. Genes from the phytosterol pathway attach to *FPPS1*, *HMGS*, *DPPS2*, *PPDS1 *and *PPDS2*.

**Figure 5:**
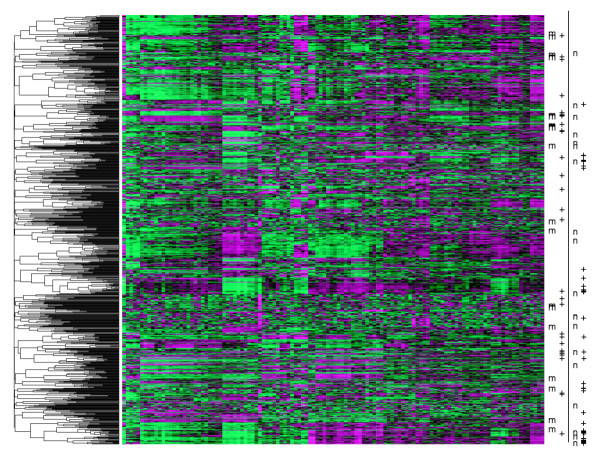
Hierarchical clustering of 40 genes involved in the isoprenoid pathway and 795 genes from other pathways. Clustering is depicted as a heatmap, in which red and green represent high and low expression values, respectively. Rows depict genes and columns depict hybridizations. Positions of the genes from the MEV pathway (m) and the plastoquinone and phytosterol pathways (+) are indicated in the left-hand column of the heatmap axis on the right side of the figure. Positions of the genes from the MEP pathway (n) and the plastoquinone, carotenoid and chlorophyll pathways (+) are indicated in the right column of the axis.

Incorporating 795 additional genes into the isoprenoid genetic network would not have been feasible with standard GGMs as the graphical model would have had to be newly fitted for each additional gene. Also, hierarchical clustering would not have been an appropriate tool for detecting the similarities in the correlation patterns between the two isoprenoid metabolisms and their downstream pathways. Figure 5 shows the hierarchical clustering of the 40 isoprenoid genes and 795 additional pathway genes based on the distance measure 1 - |*σ*_*ij*_|, where *σ*_*ij *_denotes the pairwise correlation between genes *i *and *j*.

The positions of the MVA pathway genes (labeled 'm') and the non-mevalonate pathway genes (labeled 'n'), respectively, are shown to the right of the figure. The symbol + represents the positions of genes from the downstream pathways identified in Table 2, whereby the vertical line is drawn to distinguish between genes downstream of the mevalonate and the non-mevalonate pathway. From Figure 5 it can be easily seen that there is no clear pattern of (positional) association between genes of the isoprenoid biosynthesis and downstream pathways in the hierarchical clustering.

### Simulation study

For an independent comparison between the modified and the conventional GGM approaches, we simulated gene-expression data with 40 genes and 100 observations. This simulation framework corresponds to the data for isoprenoid biosynthesis and is thought to be only exemplary at this point. An extensive simulation study is currently underway and will be presented elsewhere.

Following recent findings on the topology of metabolic and protein networks [29,30], we simulated scale-free networks in which the fraction of nodes with *k *edges decays as a power law ∝ *k*^-*γ*^. For metabolic and protein networks, *γ *is usually estimated to range between 2 and 3, which would result in very sparse networks with fewer edges than nodes in our simulation settings. To allow for denser networks, we generated 100 graphs each for *γ *= 0.5, 1.5 and 2.5. With 40 nodes, these graphs then comprised 88.3, 49.7 and 30.5 edges on average. For each edge, the conditional dependence of the corresponding gene pairs was modeled with a latent random variable in a structural equation model as described in [31]. Further details are of technical nature and are omitted here. The use of latent random variables enabled us to model partial correlation coefficients according to the previously defined network structure while ensuring positive definiteness of the complete partial correlation matrix. This matrix was then transformed into a covariance matrix Σ, from which synthetic gene expression data with 100 observations were sampled according to a multivariate normal distribution *N*(0,Σ).

**Figure 6:**
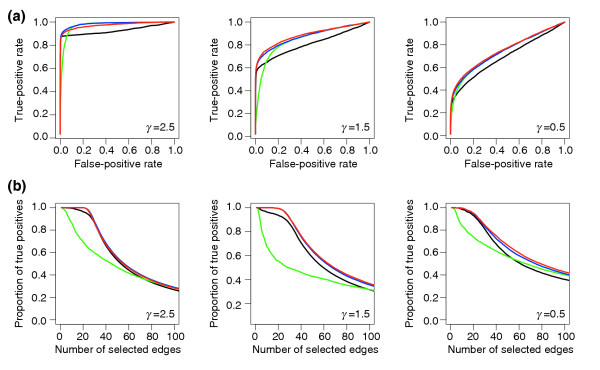
Performance of different GGM approaches. **(a) **ROC curves and **(b) **the proportion of true-positive edges as a function of the number of selected edges for the different graphical modeling strategies. Black line, the standard GGM; red line, frequentist modified GGM approach; blue line, latent random graph modified GGM approach; green line, pairwise correlation. Sparse networks with fewer edges as nodes (*γ *= 2.5) are represented in the left column, networks with approximately as many edges as nodes (*γ *= 1.5) are represented in the middle column, and networks with approximately twice as many edges as nodes (*γ *= 0.5) are in the right column.

The performance of the graphical modeling approaches was monitored using the rate of true and false positives in receiver operator characteristics (ROC) curves (see [11] for a short introduction). For the standard graphical model, bootstrapping would have been too time-consuming, so we ranked all edges according to their sequential removal in the backward selection process. Figure 6a shows the ROC curves for the graphical modeling with backward selection and the modified graphical modeling approaches (frequentist and latent random graph approach). We also included the ROC curve for network inference with pairwise correlation coefficients. It can be seen that the modified GGM approaches outperform the conventional graphical modeling. Both the frequentist and the latent random graph method show a similar performance. Also, it should be noted that a simple measure such as the pairwise correlation can be quite powerful in detecting conditional dependencies between genes.

ROC curves depict the true-positive rate as a function of the false-negative rate. However, in our setting where the false-positive edges by far outnumber the true-positive ones, the proportion of true positives among the selected edges is also of interest (Figure 6b). Note that this proportion is the complementary false-discovery rate 1-FDR [32]. Figure 6b provides further evidence that the modified GGM approaches have a better performance than standard GGM.

### Application to galactose utilization in *Saccharomyces cerevisiae*

For further evaluation, we applied our approach to the galactose-utilization dataset from [14] to detect galactose-regulated genes in *Saccharomyces cerevisiae*. Ideker *et al*. [14] used self-organizing maps to cluster 997 genes with significant expression changes in 20 systematic perturbation experiments of the galactose pathway. From the nine galactose genes under investigation, two subgroups with three and four genes, respectively, were found in two of the 16 clusters. Nine of the 87 genes in these two clusters carried GAL4p-binding sites and are thus candidate genes for regulation by the transcription factor GAL4p. Among these candidate genes, *GCY1 *and *PCL10 *are known to be targets of GAL4p [33], and *YMR318C *has been implicated in another binding-site study [34].

After incorporating all yeast genes into our network of the nine galactose genes, 13 genes were found to attach significantly well. Among these, *GCY1 *and *PCL10 *were also detected. Furthermore, three out of the remaining 11 candidate genes (*MLF3*, *YEL057C *and *YPL066W*) had GAL4p-binding sites. These genes were also identified in [14]. This result shows once more that with our approach we are not only able to model the dependence between genes but also find genes whose expression profiles fit well to the original genes in the model. In contrast to [14], we did not have to rely on gene clusters with a high occurrence of galactose genes to find these genes.

## Discussion

Analysis of gene expression patterns, for example cluster analysis, often focuses on coexpression and pairwise correlation between genes. Graphical models are based on a more sophisticated measure of conditional dependence among genes. However, with this measure, modeling is restricted to a small number of genes. With a larger set of genes, it is rather difficult to interpret the model and to generate hypotheses on the regulation of genetic networks.

In our approaches, in the search for significant co-regulation between two genes all other genes in the model are also taken into account. However, the effect of these genes is examined separately, one gene at a time. Because of this simplification, modeling can include a larger number of genes. Also, each edge has a clear interpretation, representing a pair of significantly correlated genes whose dependence cannot be explained by a third gene in the model. Our frequentist method has a resemblance to the first two steps in the SGS and PC algorithms [31]. By restricting the modeling to subnetworks with three genes, we avoid the statistically unreliable and computationally costly search for conditional independence in large subsets, as in the SGS algorithm. Also, we avoid having to remove edges in a stepwise fashion, as in the PC algorithm. Therefore, we do not run the risk of mistakenly removing an edge at an early stage, which leads to improved stability in the modeling process.

By using a Gaussian model, we can only reveal linear dependencies between genes. For handling nonlinearities, gene-expression profiles should be discretized and analyzed in a multinomial framework. In principle, it should be straightforward to adopt our approach to a multinomial model. Because we focused on linear dependencies, we have not addressed this problem so far.

For the isoprenoid biosynthesis pathways in *A. thaliana*, we constructed a genetic network and identified candidate genes for cross-talk between both pathways. Interestingly, both positive and negative correlations were found between the identified candidate genes and the corresponding pathways. *AACT1 *and *HMGR1*, key genes of the MVA pathway, were found to be negatively correlated to the module of connected genes in the MEP pathway. This suggests that in the experimental conditions tested, *AACT1 *and *HMGR1 *may respond differently (than the MEP pathway genes) to environmental conditions, or that they possess a different organ-specific expression profile. In either case, expression within both groups seems to be mutually exclusive. On the other hand, a positive correlation was identified between *IPPI1 *and members of the MVA pathway, suggesting that this enzyme controls the steady-state levels of IPP and DMAPP in the plastid when a high level of transfer of intermediates between plastid and cytosol takes place.

Although we have considered only metabolic genes in this analysis, the method can be extended to identify genes encoding other types of proteins belonging to the same transcription module. In fact, transcription factors and other regulator proteins, as well as structural proteins such as transporters, are often found in the same expression module [26]. Our results suggest that the expression of genes belonging to the chlorophyll and carotenoid biosynthesis pathways is controlled by a module that possibly includes genes from the MEP pathway.

Similarly, the expression of genes in the phytosterol pathway appears to be influenced by genes from the MVA pathway. For the downstream regulation of plastoquinone biosynthesis, however, genes from both pathways seem to be involved. This finding is in agreement with the dual localization of enzymes from the plastoquinone pathway in either the plastid or the cytosol. The regulation of this pathway may therefore depend on processes happening on the metabolic and regulatory level in both compartments.

We have shown in a simulation study that for gene-expression data with many genes and few observations, the modified GGM approaches have performed better in recovering conditional dependence structures than conventional GGM. However, a final evaluation of our inferred network for the isoprenoid biosynthesis pathways in *A. thaliana *can only be made on the basis of additional knowledge and biological experiments. At this stage, the use of domain knowledge has provided some means of network validation. As genes from the respective downstream pathways were significantly more often attached to the isoprenoid network than were candidate genes from other pathways, we are quite confident that our method can grasp the modularity in the dependence structure within groups of genes and also between groups of genes. Such modularity would have been difficult to detect by standard graphical modeling or clustering.

## Materials and methods

### Graphical Gaussian models (GGMs)

Let *q *be the number of genes in the network, and *n *be the number of observations for each gene. The vector of log-scaled gene-expression values, *Y *= (*Y*_1_,...,*Y*_*q*_) is assumed to follow a multivariate normal distribution *N*(*μ*,Σ) with mean *μ *= (*μ*_1_,...,*μ*_*q*_) and covariance matrix Σ. The partial correlation coefficients *ρ*_*ij*|*rest*_, which measure the correlation between genes *i *and *j *conditional on all other genes in the model are calculated as



where *ω*_*ij*_, *1*, *j *= 1,...,*q *are the elements of the precision matrix Ω = Σ^-1^.

Using likelihood methods, each partial correlation coefficients *ρ*_*ij*|*rest *_can be estimated and tested against the null hypothesis *ρ*_*ij*|*rest *_= 0 [5]. An edge between genes *i *and *j *is drawn if the null hypothesis is rejected. Since the estimation of the partial correlation coefficients involves matrix inversion, estimators are very sensitive to the rank of the matrix. If the model comprises many genes, estimates are only reliable for a large number of observations.

Commonly, the modeling of the graph is carried out in a stepwise backward manner starting from the full model from which edges are removed consecutively. The process stops when no further improvement can be achieved by removal of an additional edge. The final model is usually evaluated by bootstrapping to exclude spurious edges in the model.

### Modified GGM approaches

Let *i*, *j *be a pair of genes. The sample Pearson's correlation coefficient *σ*_*ij *_is the commonly used measure for coexpression. For examining possible effects of other genes *k *on *σ*_*ij*_, we consider GGMs for all triples of genes *i*, *j*, *k *with *k *≠ *i*, *j*. For each *k*, the partial correlation coefficient *ρ*_*ij*|*k *_is computed and compared to *σ*_*ij*_. If the expression level of *k *is independent of *i *and *j*, the partial correlation coefficient would not differ from *σ*_*ij*_. If on the other hand, the correlation between *i *and *j *is caused by *k *since *k *co-regulates both genes, one would expect *ρ*_*ij*|*k *_to be close to 0. Here, we use the terminology, that *k *'explains' the correlation between *i *and *j*.

In order to combine the different *ρ*_*ij*|*k *_values in a biologically and statistically meaningful way, we define an edge between *i *and *j *if *ρ*_*ij*|*k *_≠ 0 for all remaining genes *k*. In particular, if there is at least one *k *with *ρ*_*ij*|*k *_= 0, no edge between *i *and *j *is drawn since the correlation between *i *and *j *may be the effect of *k*. Our approach can be implemented as a frequentist approach in which each edge is tested for presence or absence or alternatively, as a likelihood approach with parameters *θ*_*ij*_, which describe the probability for an edge between *i *and *j *in a latent random graph.

### Frequentist approach

For the gene pair *i*, *j *and all remaining genes *k*, p-values *ρ*_*ij*|*k *_are obtained from the likelihood ratio test of the null hypothesis *ρ*_*ij*|*k *_= 0. In order to combine the different *p*-values *ρ*_*ij*|*k*_, we simply test whether a third gene *k *exists that 'explains' the correlation between *i *and *j*. For this purpose, we apply the following procedure:

(1) For each pair *i*, *j *form the maximum *p*-value

*p*_*ij*,max _= max{*p*_*ij*|*k*_, *k *≠ *i, j*}.

(2) Adjust each *p*_*ij*,max _according to standard multiple testing procedures such as FDR [32].

(3) If the adjusted *p*_*ij*,max _value is smaller than 0.05, draw an edge between the genes *i *and *j*; otherwise omit it.

The correction for multiple testing in step 2 is carried out with respect to the possible number of edges (*q*(*q *- 1))/2 in the model. Implicitly, multiple testing over all genes *k *is also involved in step 1. However, because the maximum over all *p*_*ij*|*k *_is considered, a multiple testing correction is not necessary.

### Latent random graph approach

The frequentist approach has the disadvantage that a connection between two genes *i *and *j *is either considered to be present or absent. Also, it is not taken into account whether an edge between *i *and *k *respectively *j *and *k *is truly present when we test for *ρ*_*ij*|*k *_= 0. In our second method, we introduce a parameter *θ*_*ij *_as the probability for an edge between two genes *i *and *j *in a latent random graph model. Let *θ *be the parameter vector of *θ*_*ij *_for all 1 ≤ *i *<* j *≤ *q *and *y *= (*y*^1^,...,*y*^*n*^) be a sample of *n *observations. For estimating *θ*, we maximize the log-likelihood *L*(*θ*) = log*P*_*θ*_(*y*) via the EM-algorithm [35].

Let *θ*^*t *^be a current estimate of *θ*. Further, let *g *be the unobserved graph encoded as an adjacency matrix with *g*_*ij *_∈ {0,1} depending on whether there is an edge between genes *i *and *j *or not. In the E-step of the EM-algorithm, the conditional expectation of the complete data log-likelihood is determined with respect to the conditional distribution *p*(*g*|*y*,*θ*^*t*^),



By assuming independence between edges, Equation (1) becomes



and further, after replacing



and summing out Equation 2 we find



*P*(*g*_*ij *_= 1|*y*,*θ*^*t*^) and *P*(*g*_*ij *_= 0|*y*,*θ*^*t*^) at the right side of Equation (3) are approximated by the statistical evidence of edge *i*, *j *in GGMs with genes *i*, *j *and *k*. As we only want to estimate the effect of *k *on the correlation between *i *and *j*, we distinguish only the two cases whether *k *is a common neighbor of *i *and *j*, for example, *g*_*ik *_= 1 and *g*_*jk *_= 1 or not. When *k *is a common neighbor, we test *ρ*_*ij*|*k *_≠ 0 versus *ρ*_*ij*|*k *_= 0. When *k *is not a common neighbor of *i *and *j*, we test *σ*_*ij *_≠ 0 versus *σ*_*ij *_= 0 for the pairwise correlation coefficients instead. Thus, we obtain



where  and  are *p*-values of the corresponding likelihood ratio tests. After replacing Equation (4) in Equation (3), the M-step of the EM-algorithm, that is the maximization of *E*_*θ*_(log*P*_*θ*_(*g*)|*y*,*θ*^*t*^) with respect to *θ*, leads to an iterative updating scheme *θ *^*t *^→ *θ*^*t*+1 ^with



In summary, we determine the probability parameters *θ *as follows

(1) For gene pairs *i*, *j*, compute *P*(*ρ*_*ij*_|*k *≠ 0) and *P*(*σ*_*ij *_≠ 0) for all genes *k *≠ *i*, *j*.

(2) Starting with *θ*^0^, apply iteratively Equation (5) until the error |*θ*^*t*+1 ^- *θ*^*t*^| drops below a prespecified value, for example 10^-6^.

Our latent random graph approach also enables us to fit a large number of additional genes into a constructed genetic network. In this case, for a gene pair *i*, *j *in step 1 of the analysis, the partial correlation coefficients *ρ*_*ij*|*k *_are not only computed and tested for genes *k *in the model but also for the additional candidate genes. However, the iteration in step 2 is not extended to these candidate genes. In other words, *θ*_*ij *_is only iteratively updated in Equation (5) if both genes *i*, *j *are in the original model. For candidate genes *k*, *θ*_*ik *_and *θ*_*jk *_are kept fixed at a prespecified value, for example 1, and are not re-estimated in the EM-iteration process.

This outline introduces a second level into the modeling process. At the first level, the network between the original genes is constructed. At the second level, we test how additional candidate genes influence the parameters *θ*. If these candidates have an effect on the correlation between *i *and *j*, *θ*_*ij *_will decrease. Thus, by comparing the original network with the network inferred from allowing for additional genes in step 1, we can determine which candidate genes lower the *θ*-values and, accordingly, fit well into the network.

## Additional data files

Additional data is available with the online version of this paper. Additional data files 1 and 2 contain the gene expression values of the isoprenoid genes (Additional data file 1) and the 795 genes from other pathways (Additional data file 2). Additional data file 3 contains a more detailed description of the microarray data (such as experimental conditions, hybridization and standardization). Additional data file 4 describes the correlation pattern of the 40 isoprenoid genes.

## Supplementary Material

Additional data file 1 The gene expression values of the isoprenoid genes Click here for additional data file

Additional data file 2 The gene expression values of the 795 genes from other pathways Click here for additional data file

Additional data file 3 A more detailed description of the microarray data (such as experimental conditions, hybridization and standardization) Click here for additional data file

Additional data file 4 The correlation pattern of the 40 isoprenoid genes. Click here for additional data file

## Figures and Tables

**Table 1:** Genes coding for enzymes in the two isoprenoid pathways

Name	AGI number	Subcellular location
AACT1	At5g47720	C
AACT2	At5g48230	C
CMK	At2g26930	P
DPPS1	At2g23410	C/ER
DPPS2	At5g58770	M
DPPS3	At5g58780	ER
DXPS1	At3g21500	P
DXPS2	At4g15560	P*
DXPS3	At5g11380	P
DXR	At5g62790	P*
FPPS1	At4g17190	C
FPPS2	At5g47770	C/M*
GGPPS1	At1g49530	M*
GGPPS2	At2g18620	P
GGPPS3	At2g18640	C/ER*
GGPPS4	At2g23800	C/ER*
GGPPS5	At3g14510	M
GGPPS6	At3g14530	P
GGPPS7	At3g14550	P*
GGPPS8	At3g20160	C/ER
GGPPS9	At3g29430	M
GGPPS10	At3g32040	P
GGPPS11	At4g36810	P*
GGPPS12	At4g38460	P
GPPS	At2g34630	P*
HDR	At4g34350	P
HDS	At5g60600	P*
HMGR1	At1g76490	C/ER*
HMGR2	At2g17370	C/ER*
HMGS	At4g11820	C
IPPI1	At3g02780	P
IPPI2	At5g16440	C
MCT	At2g02500	P*
MECPS	At1g63970	P
MK	At5g27450	C
MPDC1	At2g38700	C
MPDC2	At3g54250	C
PPDS1	At1g17050	P
PPDS2	At1g78510	P
UPPS1	At2g17570	M

Subcellular locations are pooled from experimental data, the TargetP data base [36] and [22]. C, cytoplasm; ER, endoplasmic reticulum; M, mitochondrion; P, chloroplast. Experimentally verified subcellular locations are marked with an asterisk (*).

**Table 2:** Pathways whose genes attach significantly well to the isoprenoid pathways

Both isoprenoid pathways	MEP pathway	MVA pathway
Plastoquinone*	Plastoquinone*	Plastoquinone*
Carotenoid*	Carotenoid*	Phytosterol*
Calvin cycle	Porphyrin/chlorophyll*	
Histidine	One carbon pool	
One carbon pool	Calvin cycle	
Tocopherol*		
Porphyrin/chlorophyll*		

Downstream pathways are marked with an asterisk (*). The Calvin cycle is also metabolically linked to the isoprenoid pathways.
