# Supplementary material for: Sparse graphical Gaussian modeling of the isoprenoid gene network in Arabidopsis thaliana
Source: Genome Biol. 2004 Oct 25;5(11):R92. doi: 10.1186/gb-2004-5-11-r92 (PMC545783; doi:10.1186/gb-2004-5-11-r92)
Supplement: Additional data file 3 — A more detailed description of the microarray data (such as experimental conditions, hybridization and standardization) [file gb-2004-5-11-r92-s3.pdf]

Description of experimental conditions

Experimental procedures involved seedlings, leaves and roots. For experiments involving seedlings or leaves, plants were grown in growth chambers at 70% humidity and daily cycles of 16 h light at 21°C and 8 h darkness at 21°C. Plant material from three independent experiments (replications not in parallel) for each experiment group respectively was pooled prior to RNA extraction. The 118 different experimental conditions are denoted by c1-c118:

c1-c2:
Experiment with wild-type and era mutant seedlings. (growth stage 1.0; tissue: whole seedlings).
c3-c5:
Arabidopsis tissue culture, leaf and seedling in a baseline experiment (see also [38]). (growth stage: - ; tissue: tissue culture, seedling, adult leaf).
c6-c14:
RNA was extracted from seedlings and adult leaves of wild-type and prenylation mutant plants grown under standard conditions (growth stage 1.0; tissue: whole seedlings and adult leaves).
c15-c22:
RNA was extracted from wild-type and several transgenic seedlings (growth stage: 1.0; tissue: whole seedlings).
c23-c30:
RNA was extracted from a root inducible system (see [39]) exposed to hormonal treatments. (growth stage: 1.0; tissue: lateral roots).
c31-c56:
Arabidopsis seedlings were exposed to light and dark conditions in a time-course experiment (0, 10 min, 1h, 5h, 2d, 5d). (growth stage: 1.0; tissue: whole seedlings).
c70-c92:
Experiment to assess the effect of inhibitors of the MVA pathway (lovastatin) and the MVA-independent pathway (fosmidomycin) on the expression of genes involved in isoprenoid biosynthesis. (growth stage: 1.0 and 3.90; tissues: whole seedlings and adult leaves).
c93-c118:
Arabidopsis seedlings and adult leaves were exposed to ozone for several periods of time. (growth stage: 1.0 and 3.90; tissues: whole seedlings, cauline leaves and adult leaves).

Oligonucleotide arrays:
Affymetrix Arabidopsis ATH1 GeneChip® arrays (Affymetrix, Santa Clara, California; [40]) were used throughout the experiments.

RNA purification:
Total RNA was prepared from frozen seedling and leaf tissue using Trizol and purified with RNeasy columns (Quiagen, Hilden, Germany). Fifteen micrograms of total RNA were used to prepare cDNA with the Superscript Double-Stranded cDNA Synthesis Kit (Invitrogen) according to manufacturers instructions using oligodT-T7 oligonucleotides (GGCCAGTGAATTGTAATACGACTCACTATAGGGAGGCGG(dT)24). The cDNA was subjected to in vitro transcription in the presence of 2mM each of biotin-11-CTP and biotin-16-UTP (ENZO Life Sciences, Farmingdale, NY) using the MegaScript High Yield Transcription Kit (Ambion, Austin, TX). After purification of the cRNA on RNeasy columns (Qiagen, Hilden, Germany), 15mg cRNA were fragmented in a volume of 40ml as recommended by Affymetrix.

Hybridization:
The fragmented 15mg of labeled cRNA were denatured for 5min at 99°C and hybridized to the arrays for 16 hrs as recommended by Affymetrix. Washing and detection of labeled cRNA using streptavidin-phycoerythrin were performed according to manufacturer's instructions using the EukGE-WS2v3 protocol

involving two streptavidin-phycoerythrinlabelling steps.

Scanning:
The arrays were scanned using a confocal scanner Agilent GS 2500.

Normalization:
Raw data was processed with the statistical algorithm of Affymetrix Microarray Suite 5.0 as described [41] using the default parameters. Each array was normalized to a target value (TGT) of 1000.
