# Supplementary material for: Sparse graphical Gaussian modeling of the isoprenoid gene network in Arabidopsis thaliana
Source: Genome Biol. 2004 Oct 25;5(11):R92. doi: 10.1186/gb-2004-5-11-r92 (PMC545783; doi:10.1186/gb-2004-5-11-r92)
Supplement: Additional data file 4 — The correlation pattern of the 40 isoprenoid genes. [file gb-2004-5-11-r92-s4.pdf]

Supplementary Figure 1

Pairwise correlation coefficients between the 40 isoprenoid genes. Genes were ordered with respect to "similarity" in their correlation patterns based on the first two principal components [37].

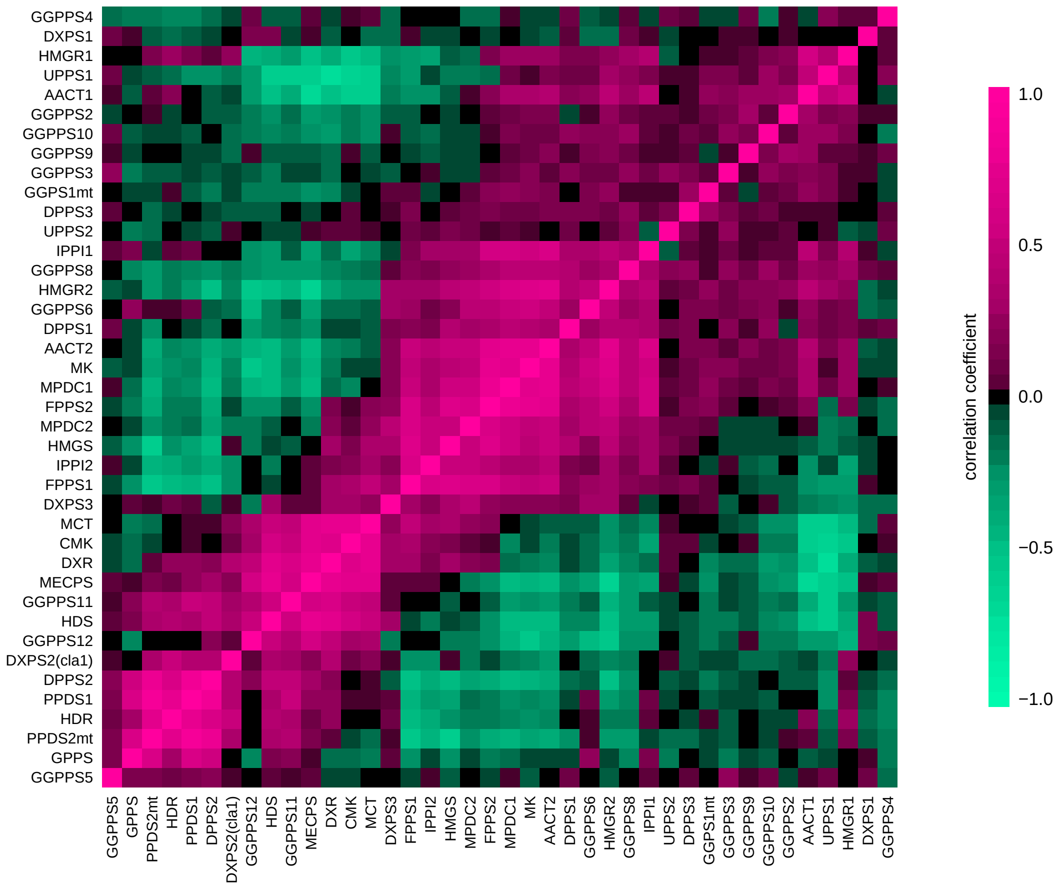

The correlation patterns of the 40 isoprenoid genes display several overlapping blocks of positively correlated genes. These gene blocks are partly reflected in our inferred graphical model for the two isoprenoid pathways. For example, the genes DXR, MCT, CMK, MECPS, HDS, GGPPS11, GGPPS12 are all positively correlated with each other. From these gene block, the genes DXR, MCT, CMK, MECPS were later identified to form a module of densely connected genes in the graphical model. However, the gene GGPPS12 was connected to the genes HMGR2 and MK in the MVA pathway.
